# Artificial Cornea: Past, Current, and Future Directions

**DOI:** 10.3389/fmed.2021.770780

**Published:** 2021-11-12

**Authors:** Gráinne Holland, Abhay Pandit, Laura Sánchez-Abella, Andrea Haiek, Iraida Loinaz, Damien Dupin, Maria Gonzalez, Eva Larra, Aritz Bidaguren, Neil Lagali, Elizabeth B. Moloney, Thomas Ritter

**Affiliations:** ^1^School of Medicine, College of Medicine, Nursing and Health Sciences, Regenerative Medicine Institute, National University of Ireland Galway, Galway, Ireland; ^2^CÚRAM Science Foundation Ireland Research Centre for Medical Devices, National University of Ireland, Galway, Ireland; ^3^CIDETEC, Basque Research and Technology Alliance, Parque Científico y Tecnológico de Gipuzkoa, Donostia-San Sebastián, Spain; ^4^AJL Ophthalmic, Alava, Spain; ^5^Ophthalmology Department, Donostia University Hospital, San Sebastián, Spain; ^6^Department of Biomedical and Clinical Sciences, Faculty of Medicine, Linköping University, Linköping, Sweden

**Keywords:** artificial cornea, blindness, biointegration, Boston type-1 keratoprostheses, osteo-odonto-keratoprostheses, AlphaCor™, keratoprosthesis, bio-mimetic cornea

## Abstract

Corneal diseases are a leading cause of blindness with an estimated 10 million patients diagnosed with bilateral corneal blindness worldwide. Corneal transplantation is highly successful in low-risk patients with corneal blindness but often fails those with high-risk indications such as recurrent or chronic inflammatory disorders, history of glaucoma and herpetic infections, and those with neovascularisation of the host bed. Moreover, the need for donor corneas greatly exceeds the supply, especially in disadvantaged countries. Therefore, artificial and bio-mimetic corneas have been investigated for patients with indications that result in keratoplasty failure. Two long-lasting keratoprostheses with different indications, the Boston type-1 keratoprostheses and osteo-odonto-keratoprostheses have been adapted to minimise complications that have arisen over time. However, both utilise either autologous tissue or an allograft cornea to increase biointegration. To step away from the need for donor material, synthetic keratoprostheses with soft skirts have been introduced to increase biointegration between the device and native tissue. The AlphaCor™, a synthetic polymer (PHEMA) hydrogel, addressed certain complications of the previous versions of keratoprostheses but resulted in stromal melting and optic deposition. Efforts are being made towards creating synthetic keratoprostheses that emulate native corneas by the inclusion of biomolecules that support enhanced biointegration of the implant while reducing stromal melting and optic deposition. The field continues to shift towards more advanced bioengineering approaches to form replacement corneas. Certain biomolecules such as collagen are being investigated to create corneal substitutes, which can be used as the basis for bio-inks in 3D corneal bioprinting. Alternatively, decellularised corneas from mammalian sources have shown potential in replicating both the corneal composition and fibril architecture. This review will discuss the limitations of keratoplasty, milestones in the history of artificial corneal development, advancements in current artificial corneas, and future possibilities in this field.

## Introduction

**Figure 1:**
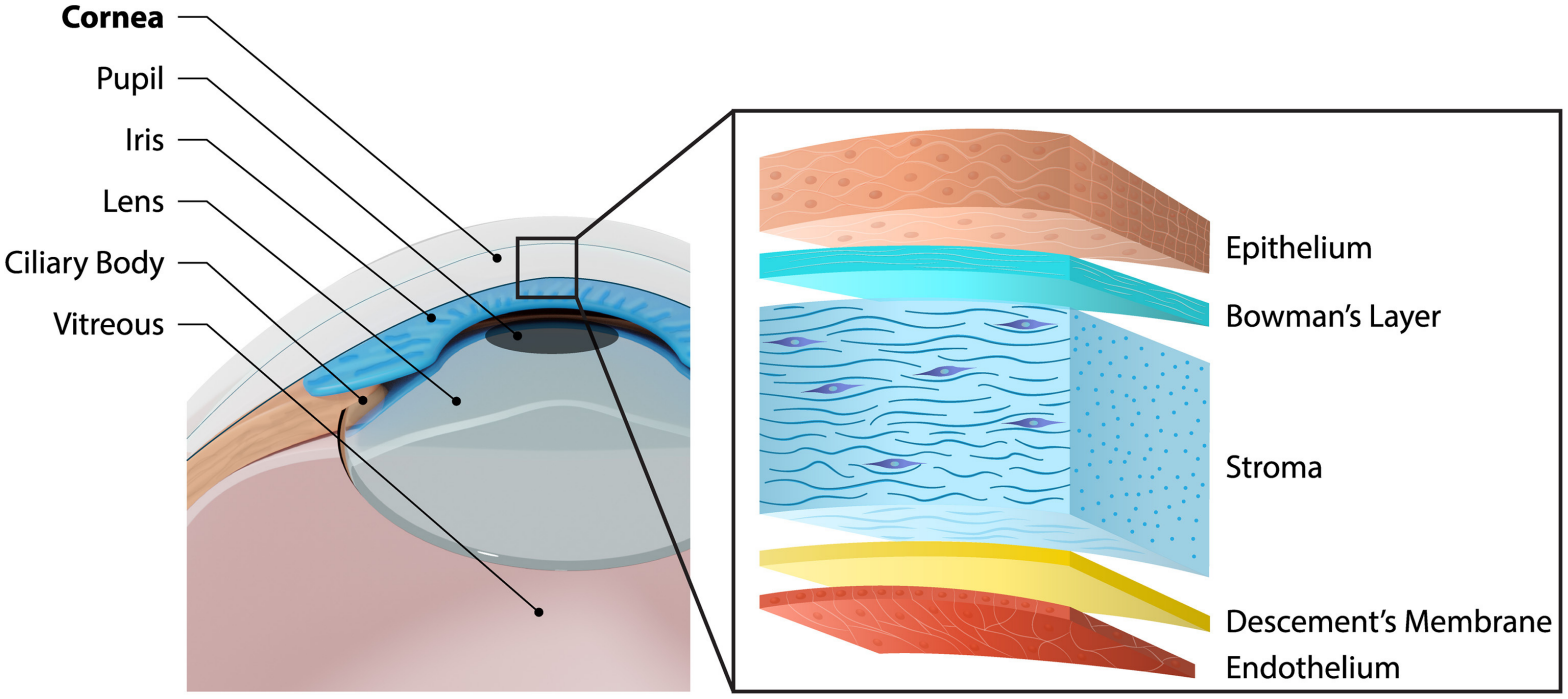
Schematic diagram of the anatomical position of the cornea, and its 5 distinguishable layers.

Located at the front of the eye, covering the pupil, iris, and anterior chamber, the cornea is the primary component of the ocular optical system (1). The cornea is made up of three cellular layers- epithelium, stroma, and endothelium; and two acellular layers- Bowman's and Descemet's membranes (Figure 1) (2). The outermost layer, the epithelium, which makes up 10% of the total corneal thickness, consists of stratified cells with tight junctions, that form a protective barrier. Between the epithelium and stroma is the Bowman's layer, an acellular layer often known as a modified extension of stroma (3). The stroma makes up 90% of the total corneal thickness. It protects the eye from the external environment while contributing to 65–75% of all light transmission to the retina, enabling vision. Separating the posterior corneal stroma and endothelium is the Descemet's membrane, which is a dense, thick, somewhat transparent, cell-free matrix (4). For those with corneal melting disorders, the Descemet's membrane is sometimes the only layer remaining to keep the eye's integrity. The endothelium is made up of a single layer of hexagonal cells. The function of the endothelium is to regulate and maintain stromal hydration (3). Collectively, the cornea is a highly complex tissue, innervated and avascular.

Corneal diseases are a leading cause of blindness with an estimated 10 million patients diagnosed with bilateral corneal blindness worldwide (5). Furthermore, corneal blindness affects proportionally more children and young adults than any other age-related blinding disease such as macular degeneration (6). Therefore, corneal transplantation, or keratoplasty, is the most common transplant performed globally, with ~185,000 corneal transplants performed every year in 116 countries. Unfortunately, around 1 in 70 patients or 12.7 million people are still awaiting corneal transplantation, given that the demand for donor material far exceeds the supply. This emphasises the need for an innovative solution to supplement the supply of transplantable or implantable tissues for corneal replacement, whether with bio-mimetic or artificial corneas (7).

Artificial corneas can be defined as laboratory-made constructs, with or without the help of biological material but typically consisting of manmade materials, designed principally to replace the function of the native human cornea. Typically, keratoprostheses fall into this category. The benefits of artificial corneas tend to outweigh the disadvantages (Table 1), especially in difficult and high-risk cases where traditional donor cornea transplantation would have a poor outcome. Production of keratoprostheses (or KPros) is stringent in order to guarantee non-toxic, sterile products with high stability. Moreover, KPros overcome socio-cultural and policy difficulties while preventing viral invasion and immune rejection. These KPros are specialised constructs with limited swelling which results in limited water accumulation and less light scattering from the cornea. Improvement in KPro design is possible due to the continually evolving biomaterial technologies, that enable functionalisation using synthetic materials or surface coating techniques. In addition, reservoir systems such as micro- or nanoparticles can be incorporated into these systems to facilitate biointegration and modulate inflammation. Furthermore, 3D fabrication methods can build a fully functionalised biosynthetic cornea with programmed spatial, optical properties and biomechanical properties which cannot be provided by a human corneal transplant (8).

**Table 1:** Advantages and disadvantages of keratoprostheses.

**Advantages**	**Disadvantages**
Can restore meaningful vision in the most severe cases of corneal blindness where donor corneas fail	Uncomfortable to wear
Avoids religion, culture and policy problems	Transplantation process is complex; multiple surgeries and long-term topical medications often required
Overcomes immune rejection, immune graft risk and ocular surface disease	Limited field of view
Continuously evolving technologies	Unsatisfactory aesthetic appearance
Limited swellability therefore limited water accumulation and less light scattering	Potential for post-operative complications such as extrusion and glaucoma.

### Keratoplasty and Its Limitations

Throughout the years, keratoplasty has proven to be one of the most successful transplant procedures. For low-risk patients, corneal transplantation is an attractive solution with high success rates: survival of first-time grafts is ~90% at 5 years (9, 10). However, these success rates steadily decrease over time (11). Anshu et al. investigated over one thousand penetrating keratoplasties performed over 20 years and found that corneal grafts remained in only 55.4% of patients at 10 years, 52% at 15 years and 44% at 20 years post-surgery (12). Similarly, the Australian Corneal Graft Registry (ACGR) reported that after 15 years corneal graft survival rates had dropped to 46% for full-thickness grafts and 41% for lamellar grafts (13).

Keratoplasty has proven to work with several conditions, for example, keratoconus, corneal opacities, and bullous keratopathy. However, those with recurrent or chronic inflammatory disorders such as sicca disease states, history of glaucoma and herpetic infections, and those with neovascularisation of the host bed have a low keratoplasty success rate (6, 14, 15). Furthermore, those who have failed their first keratoplasty have a high chance of re-graft failure with about 50% of re-grafts failing at 5 years (9, 16). Given this, surgeons are likely to only give re-grafts to patients with a high chance of graft survival and visual acuity improvement (6).

The need for corneal donor tissue is especially prevalent in developing countries. It is often difficult to meet the required logistics around corneal donor transplantation involving processing, transportation, and storage in developing countries due to a lack of facilities. Additionally, biological tissue can transmit certain infections such as tuberculosis, hepatitis C, and venereal infections. Although artificial corneas, or KPros, can potentially address certain limitations of keratoplasty, at present artificial corneas are not seen as an alternative, but more as a last resort. Currently, artificial corneas are only used in end-stage corneal blindness associated with a severe ocular surface disease or as a result of multiple conventional transplantation failures (14). However, advances in KPro technology may lead to KPros being chosen over keratoplasty in the future.

### History and Development of Keratoprostheses

Transplantation, including that of corneas, was first referenced around 2000 BC by the Egyptians (17). In 1760, the grandfather of Charles Darwin, Erasmus Darwin, first suggested the removal (trephination) of an opaque cornea and the addition of a KPro to restore vision (18). This was followed by the first full description of a KPro by Guillaume Pellier de Quengsy in 1789 in his monograph on ophthalmology: he suggested a thin silver-rimmed convex glass disc can be used in place of an opaque cornea with the surgical instruments required for such a procedure (19).

Nonetheless, there was little interest in KPros at that time. In 1853, Nussbaum manufactured a quartz crystal and implanted it into the cornea of rabbits. The first prototype was too large and was rapidly extruded. However, a smaller oblong-shaped prototype was successful in animals and was tried in human patients. These initial KPros had a high failure rate due to infection, leakage, and extrusion of the device (20). Six years later, Heusser successfully implanted a quartz KPro into a blind girl's cornea in Switzerland, who experienced a significant improvement in vision and retained the implant for at least 6 months without complications (21).

In 1862, Abbate made a KPro out of a glass disc surrounded by two rings assembled from natural polymers; gutta-percha and casein. The former was isolated from the exudate of trees and the latter from the precipitation of milk or cheese. The KPro implanted in cats and dogs were only retained for about a week. Although this KPro was quickly extruded, Abbate did emphasise the need for the KPros to be different from glass to allow for incorporation into the host tissue (3, 14). In the early twentieth century, Salzer implanted a quartz disc bounded by a platinum ring with prongs into four humans, with one almost lasting 3 years (22). Much like Abbate, Salzer suggested later that the rim of the KPro should be based on materials that can be incorporated into the host cornea. He also noted that KPros could be made out of materials lighter than glass (3).

Investigation into KPros stalled after Eduard Zirm performed the first successful bilateral keratoplasty in 1905 (23). However, the discovery of poly (methyl methacrylate) (PMMA) in WWII by Harold Ridley refocused attention on artificial corneas. PMMA splinters from crashed Perspex^®^ canopies were found embedded in the cornea of pilots' eyes and were observed to be well-tolerated, thus providing a potential material for subsequent KPros (20, 24). To date PMMA has proven to be the material of choice, providing a stable and minimally toxic optic. Over time, a two-part “core–skirt” structure was devised for the KPro. Stone took advantage of PMMA to make perforated discs and implant them in corneal lamellae, which were retained for 3 years on average (25, 26). Using a PMMA skirt positioned retrocorneally forming the “nut,” and the threaded optic making up the bolt, Cardona developed the first two-piece nut-and-bolt KPro in 1969 (27). Five years later, Dohlman introduced the collar-button model which had a front and backplate made out of PMMA (28). Aquavella et al. performed a retrospective analysis of implanted Cardona and Dohlman devices, concluding that, although improved device design and surgical procedures reduce the severity of complications, further refinements aimed at KPro biointegration will enhance the long-term clinical outcome for patients (29).

Several scientists took inspiration from Cardona's nut-and-bolt device but used several different materials as skirts to support biointegration into the host tissue, e.g., Proplast, Teflon, hydrogels, poly-2-hydroxyethyl methacrylate and silicone-carbon (30–32). In this review, various soft and hard KPros and their design, outcomes and recent advances will be reviewed.

## Hard Keratoprostheses

Hard keratoprostheses include those made from PMMA as it is a rigid polymer that needs a resilient skirt material to function as a successful implant (Table 2). Moreover, the bonding between PMMA and its skirt must withstand intraocular pressure, and deformations caused by movement of the eye and blinking. Therefore, skirts made from softer materials like Dacron, Teflon and Proplast were extruded (30–32). KPro models like the Boston KPro and osteo-odonto-keratoprosthesis (OOKP), based on harder skirts have been successful in wet blinking eyes and dry or non-blinking eyes, respectively. Although other hard KPros exist, such as the Fyodorov-Zuev KPro (40, 41), this review will focus on the Boston KPro and OOKP as there is an abundance of literature to demonstrate their efficacy in restoring sight, as well as a multitude of studies documenting improvements to their design and/or surgical procedure for enhancing clinical outcomes.

**Table 2:** Description of commercial Hard-Keratoprostheses with skirts based on resilient materials, and transparent optic cylinders composed of polymethyl methacrylate (PMMA).

**Keratoprosthesis**	**KPro materials**	**Schematic**	**References**
Cardona keratoprosthesis	Teflon (skirt) PMMA (optic)	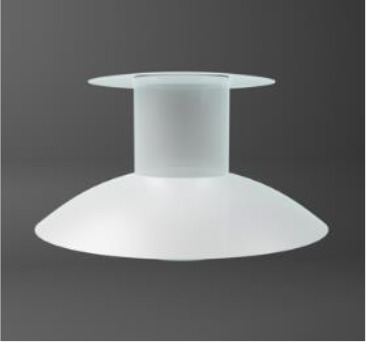	(29, 30)
Boston Keratoprosthesis [type I and type II]	Titanium (skirt) PMMA (optic)	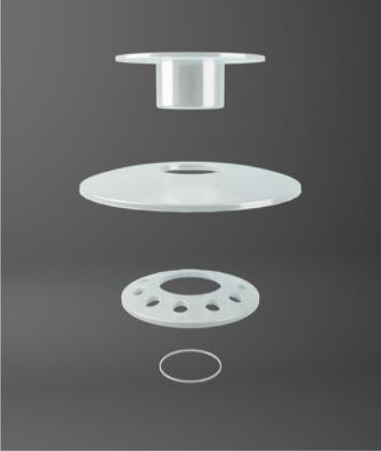	(33–35)
The osteo-odonto-keratoprosthesis (OOKP)	Autologous tooth root and alveolar bone PMMA (optic)	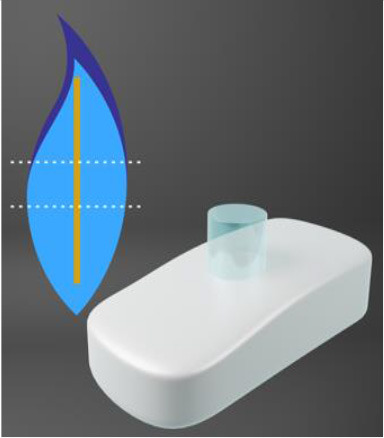	(36, 37)
The modified osteo-odonto-keratoprosthesis (MOOKP)	Osteodental lamina surface PMMA (optic)	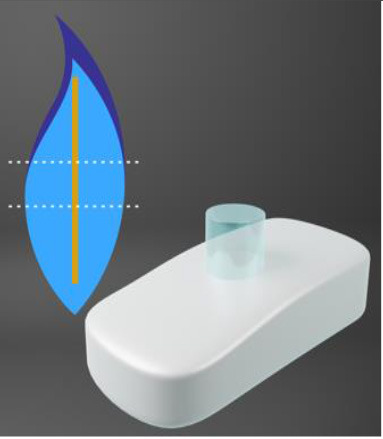	(38, 39)
Fyodorov-Zuev keratoprosthesis (MICOF)	Titanium (skirt) PMMA (optic)	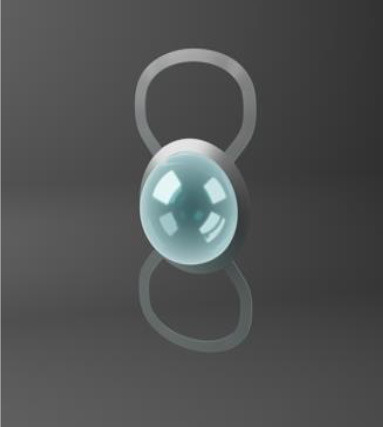	(40, 41)

### Boston Keratoprosthesis

As mentioned previously, the collar-button model called Dohlman–Doane KPro was a predecessor to the Boston KPro (28). In 1992, the type-I Boston KPro was approved by FDA. Since then it has become the most implanted KPro with over 15,000 devices implanted worldwide. The type II Boston KPro is less popular than its counterpart and is indicated for patients with severe ocular diseases, for example, Stevens-Johnson syndrome (SJS) and mucous membrane pemphigoid (MMP) (33). The main difference consists in the anterior extension which allows implantation through surgically closed eyelids (34).

#### Design

The Boston KPro consists of a PMMA front plate with a central diameter between 3.5 and 3.7 mm and a backplate made of PMMA or titanium. A titanium locking ring was also added to secure the backplate (42). Donor corneal tissue acts as a carrier and is placed between the front and backplate. It was found that both frozen and fresh donor corneas could be used for Boston KPro Type 1 (43). It is thus important to consider that these KPros do not eliminate the need for donor human corneas but work in tandem with donor corneas.

#### Outcomes

The majority of short-term outcomes of the Boston KPro type-I are favourable. Retention rates are around 90% with post-operative visual acuity (VA) of 20/100 or better in 67% of patients at 6 months and 75% at 1 year (35, 44). However, there is inadequate medium and long-term follow-up data following Boston KPro surgery. This is particularly true for long-term (>5 years) outcomes; both retention rates and complication data are scarce (45). It is important to know the medium- and long-term outcomes as it gives a realistic perspective on the real performance of KPros.

The majority of patients improve their VA following implantation. A meta-analysis of 406 articles found that 60% of patients had 6/60 vision or better at 2 years and 51% at 5 years (45). Kanu et al. found 75 and 66.7% of patients had improved VA at 5 and 10 years, respectively (46). Often, VA improves the longer the KPro has been implanted. Aravena et al. (47) found at 5 years 57% of patients had a corrected distance visual acuity (CDVA) ≥20/200 while at 8 years 82% of patients had a CDVA ≥20/200, while 5% of patients had a CDVA ≥20/200 pre-operatively. In contrast, Szigiato et al. (48) found only 36.5% of patients had a VA ≥20/200 post-operatively with 2.4% having a VA ≥20/200 pre-operatively. Szigaiato's study had more patients than Aravena's however (58 vs. 85). Interestingly, Driver et al. investigated 231 eyes: 67 primary KPro procedures and 164 after a failed keratoplasty. They found 78–87% of primary KPro procedures had a CDVA of ≥20/200 after 6 years. In comparison, 56–67% of those given the Boston KPro after failed keratoplasty had a CDVA of ≥20/200 at 6 years (49).

Those with inflammatory diseases like SJS have a high probability of gaining a CDVA of ≥20/200. One study found that 100% of patients with SJS had a CDVA of ≥20/200 after a year (50). Similarly, Brown et al. found 100% of patients with the herpes simplex virus (HSV) had a best-corrected visual acuity (BCVA) ≥20/200. However, only one patient out of four with herpes zoster virus (HZV) had a BCVA ≥20/200 (51). Interestingly, a study investigating patients with chemical or thermal injuries found that after a follow-up of 40.7 months on average, the median best-corrected visual acuity was 20/60 (52).

In general, retention rates for the Boston KPro have been quite high (between 74 and 100%) at the last follow-up (45, 51). The aforementioned meta-analysis review found accumulated retention rates of 88 and 74% at 2 and 5 years, respectively (45). However, conditions that cause cicatrisation like SJS or MMP can significantly decrease retention rates (50, 53). Alexander et al. (50) found an increase in post-operative complications for those with SJS which resulted in decreased retention rates. Brown et al. found a similar disparity between the HSV and HZV groups in retention rates as seen with the BCVA. The HSV group had a retention rate of 100% whereas the HZV had a retention rate of 25% after around 50 months (51). Phillips et al. (52) found that patients with chemical or thermal injuries, had an initial retention rate of 77.7% and the remaining KPros were successfully replaced. They did find that for those with severely damaged eyes, the rate of success can be increased by preparing the ocular surface before implantation with limbal stem cell transplants to reduce sterile ulceration.

#### Post-operative Complications and Advances

Adjustments in the design of the KPro were introduced to decrease post-operative complications such as the addition of holes to the backplate of the device. The backplate was originally a solid 8 mm PMMA plate which led to high keratolysis and decreased nutritional flow. Keratolysis is defined as the “thinning of peripheral corneal stroma with an overlying epithelial defect due to autoimmune-induced inflammation” (54). Currently, the backplate is 8.5 mm with 16 holes for nutritional support. This led to a decrease in keratolysis from 50 to 10% following transplantation (42). Wearing a large diameter soft or contour contact lens and long-term use of topical antibiotics also decreased sterile keratolysis (14). In 2014, a titanium backplate was introduced as an alternative to PMMA which clicks into the stem without the need for a locking ring, thus easier to assemble. Titanium is well-tolerated by the surrounding tissue and is highly resistant to corrosion and is both light and strong. As it is not magnetic, patients can undergo magnetic resonance imaging (42). Moreover, the titanium backplate can be coloured blue or brown by electrochemical anodisation to help with the cosmetic appeal of the device (55).

There are conflicting reports about whether titanium can cause a reduction in retroprosthetic membrane (RPM) formation, which occurs when fibrovascular tissue grows behind the device. Up to 65% of patients with a Boston KPro form an RPM (56). A study by Todani et al. (57) investigated the potential for RPM formation in 55 eyes with PMMA backplates and 23 with titanium backplates: 41.8% of patients with PMMA backplates developed RPM compared to only 13% for patients with titanium backplates at 6 months post-implantation. However, in the group of patients with PMMA backplates, 39 had threaded PMMA backplates which may, in itself, increase RPM formation (discussed below) (57). In contrast, a study by Talati et al. (58) compared 20 patients with a titanium backplate and 20 with a PMMA backplate with an average follow-up duration of 28.1 or 53.7 months, respectively: 45% of patients with a PMMA backplate developed RPM and 55% of those with a titanium backplate developed RPM. It was concluded that neither material was superior in reducing RPM formation.

In 2007, a newer PMMA stem was produced without screw threads. It was aimed at avoiding damage to the corneal graft associated with the screwing action during surgery and thus possibly reduce RPM formation (59). This newer stem was both easier to use and less expensive to produce as the device was produced by moulding as opposed to machine-made (42). Al Arfaj and Hantera investigated four eyes that underwent Boston type 1 threadless KPro implantation and found no RPM developed at the time of follow-up (i.e., up to 11 months) (60). This is consistent with the observations made by Todani et al. at 6 months post-surgery: 46.1% of eyes implanted with threaded PMMA backplates resulted in RPM, while RPM occurred only in 31.2% of cases implanted with threadless PMMA backplates (57). Thus, combining a PMMA backplate with a threadless design may reduce the risk for RPM formation (57).

One of the difficulties encountered by many hard KPros is the failure of the corneal graft to adhere to the surface of the PMMA stem. Although PMMA is minimally toxic to corneal stromal cells, poor biointegration between the PMMA and the corneal stroma can lead to corneal melting and graft detachment. Weak interfacial adhesion can create spaces into which bacteria or inflammatory cells can infiltrate (61). In recent years it has been demonstrated that contact between cells and titanium results in increased growth of corneal limbus epithelial cells, alongside a decrease in cell death, thereby providing a superior surface for adhesion (62, 63). Titanium with smooth surface topography was found to enhance cell adhesion and proliferation while roughened titanium can reduce vision-impairing light reflectivity (62).

Several novel techniques have been introduced recently to increase PMMA and corneal tissue adhesion. Sharifi et al. (64) used magnetron sputtering of titanium onto the Boston KPro PMMA stem to show that titanium sputtering can cause an increase in cell adhesion, with an increase in cell growth and collagen deposition, resulting in a more normal corneal stromal cell phenotype. For these reasons, titanium sputtering may improve PMMA-corneal tissue adherence, therefore improving long-term outcomes. Coating the titanium of the KPro with hydroxyapatite (HAp), a constituent from bone and teeth has also resulted in enhanced tissue adherence in rabbit corneas (65, 66). HAp nanoparticles can also be trapped and immobilised on the PMMA surface which results in human corneal fibroblasts adhering and proliferating onto the coated PMMA (61). Similarly, calcium phosphate (CaP) was used to coat PMMA sheets that had dopamine present to induce CaP deposition (61). This resulted in better adhesion, but delamination occurred rather easily. Furthermore, L-3,4-Dihydroxyphenylalanine (L-DOPA) can be covalently bonded to the PMMA surface to support enhanced cellular adhesion, proliferation, and migration, thus improving the compatibility of PMMA (67).

Another post-operative complication that may lead to possible changes in the Boston KPro design is glaucoma. Nonpassopon et al. gathered information from several Boston KPro clinical trials and found 20.2–40% of eyes had an increase in intraocular pressure (IOP), 14–36% developed *de-novo* glaucoma, and 13–33% had progression of previously present glaucoma (42). These results are primarily due to the device being unable to detect elevated IOP early with standard tonometry techniques due to the rigidity of the KPro device (68). Therefore, a potential solution has been introduced by integrating a micro-optomechanical pressure sensor into the Boston KPro device. Hui et al. investigated a fibre-optic Fabry-Perot pressure sensor for its cost-effectiveness and industrial quality control (69). The sensor integrated onto the KPro was stable over long periods and successfully measured IOP. However, pressure sensors implanted in rabbit eyes showed an increase of IOP following RPM formation. It was concluded that RPM formation shortened the optical cavity and caused an artificial IOP increase (69). Another alternative is to use three-dimensional (3D) spectral-domain optical coherence tomography (OCT) to enhance the evaluation of KPro patients with glaucoma (68).

#### Cost

In developing countries, the cost of the Boston KPro device can be prohibitive. In 2011, the Aurolab in Madurai, India designed a low-cost version of the Boston KPro, the auroKPro. Basu et al. (70) compared both KPros and found them to be similar in retention rates (70.5 vs. 62.5% for Boston Kpro and auroKPro, respectively) and post-operative complications, but more extrusions were observed with the auroKPro. In 2012, the Boston KPro team also produced a less expensive KPro, the Lucia KPro, which was approved by the FDA in 2019 (71, 72). This device had a titanium backplate which was 7.5 mm in diameter with radial petaloid-shaped holes. It was anodised to a brown colour giving a more acceptable appearance to patients. Although efforts are being made towards reducing device manufacturing cost while maintaining ease of implantation, it is important to note that there are many additional costs associated with any corneal procedure, including access to additional clinical resources, and continued post-operative care, and it is ultimately these factors that create a cost-prohibitive option for many patients requiring corneal replacement (70, 73–75).

### Osteo-Odonto-Keratoprosthesis

First introduced by Strampelli in 1963, OOKP is one of the longest-lasting KPros available (36). Strampelli used a donor root tooth and alveolar bone to support the PMMA optical cylinder (37). This was further improved upon by Falcinelli in 1998 by adding certain modifications such as using a larger biconvex optic and performing cryo-extraction of the lens. This led to the model which is now known as the modified osteo-odonto-keratoprosthesis (MOOKP) (38). The MOOKP is a device that uses the alveo-dental lamina of a single tooth (usually canine) to support the optical cylinder in its centre. This is covered with a resistant membrane called the buccal mucosa (BM) to give protection and nourishment. It is indicated for patients with bilateral corneal blindness with severe visual loss (<6/60) and dry eye or lid damage, as well as poor keratoplasty prognosis. Those with SJS, MMP, chemical or thermal injury view the OOKP as a life-changing surgery (76), and over the last 40 years, centre-based studies across Europe and India have demonstrated excellent anatomical retention of the MOOKP and improvements in visual acuity for the almost 500 patients studied (73).

#### MOOKP Device Preparation and Implantation

Creating the MOOKP device requires a complex surgical technique and patient counselling, and can only be performed by experienced surgeons. The technique can be separated into two stages: first preparing the bulbar anterior surface and the osteo-odonto-acrylic lamina, and secondly implanting the lamina OOKP into the eye (39, 76).

For those with normal conjunctiva, a 360-degree limbal peritomy is performed, followed by a superficial keratectomy to remove the epithelium and any scar tissue present (76). Oral mucosa is harvested from below the parotid duct and sutured in place, covering the cornea and sclera (39, 76). To prepare the osteo-odonto-acrylic lamina, a monoradicular tooth and surrounding alveolar bone are removed. Through constant irrigation with a balanced salt solution and with the aid of a dental flywheel, the tooth and alveolar bone are shaped into a 3 mm thick rectangular lamella. A hole is then drilled perpendicularly into the lamina to accommodate an optical cylinder. The PMMA optical cylinder is made up of an anterior stem that ranges in diameter from 3.5 to 4 mm and a posterior section ranging from 4.5 to 5.25 mm in width. The anterior stem protrudes 2–3 mm beyond the alveolar side while the posterior projects through the anterior chamber (76). The completed osteo-odonto-acrylic lamina is then inserted below the lower orbital rim under the skin for ~3 months (39).

In stage 2 the lamina is retrieved from the lower orbital rim and excess soft tissue is removed leaving new vascularisation intact. The mucosal graft is partially detached from top to bottom and the Flieringa ring is placed on the sclera to facilitate attachment of the lamina. A full-thickness disc is made in the cornea to facilitate the optical cylinder of the KPro. A 360-degree iridectomy is performed to remove the iris. This is followed by cryo-extraction of the lens. The lamina is sutured to the sclera and remaining cornea and is covered by the flap of the oral mucosa. Generally, a cosmetic prosthesis is applied which covers the ocular surface, 1 month after surgery (39). Topical broad-spectrum antibiotics must be applied every night for the patient's lifetime (76).

For those with no suitable teeth, a tooth allograft from a related or non-related donor can be used or tibial bone can be used. However, functional survival rates of KPros using tibial bone can be as low as 19% after 10 years (77). Retention of bone strength is reliant on physical stress and so inactivity leads to resorption of the laminae (78). Furthermore, a KPro has been developed for patients with unsuitable teeth for OOKP and no healthy eyelid skin for the Boston KPro type II called the “Lux” KPro (79). It is made up of a PMMA optic, titanium backplate, and a titanium sleeve but it requires a corneal graft. Like the MOOKP, the “Lux” KPro is implanted through, and protected by, a mucous membrane graft (79).

#### Outcomes

The VA of patients following OOKP surgery can be as good as 6/14. In a systematic review of eight different case studies, Tan et al. found VAs of ≥6/18 in 52% of patients after OOKP (80). Similarly, Liu et al. recorded a VA of ≥6/12 in 53% of all OOKP patients. In the same study, 78% of patients achieved a VA of ≥6/60 (81). Iyer et al. recorded 66% of all patients had a VA ≥20/60 (82). However, complications involving the mucosa, retina, lamina and IOP can occur which affect visual outcomes.

Long- and medium-term anatomical retention rates for OOKP devices are high throughout several studies. Iyer et al. found 96% retention in 50 eyes, with a mean follow-up of 15.4 months (82). Liu et al. reported 72% of patients retained their OOKP after a mean follow-up of 3.9 years (81). De la Paz et al. found 86% of patients with a chemical injury retained their OOKP while only 65% was retained when the Boston KPro type-I was used (83).

#### Post-operative Complications

A common cause of OOKP retention failure is resorption. Although there must be a balance between resorption and reformation to preserve the lamina, the osteo-odonto-acrylic lamina is prone to excessive resorption. In a study undertaken by Liu et al., 19% of patients had laminar resorption, resulting in retention failure (81). However, laminar resorption rates are most likely underreported as it tends to progress slowly and is difficult to detect as the lamella resides underneath the oral mucosal membrane graft. Laminar resorption can result in thinning of the lamina which may cause tilting of the optical cylinder, altered refraction, leaking, and endophthalmitis (76).

Advances in imaging have resulted in earlier detection of laminar resorption. Avadhanam et al. (84) found that 40% of all cases of laminar resorption were detected in the first year of follow-up and 66% of cases were found within 3 years of OOKP surgery. They also discovered that laminar thickness did not affect the onset or progression of resorption (84). Multi-detector computerised tomography (MDCT or CT) is widely employed when investigating laminar resorption. Along with imaging, clinical palpation can be carried out by an experienced surgeon to detect resorption early. It seems the best way forward is to implement both methods in the long-term. However, frequent CT scanning is not indicated for the detection of laminar resorption (85).

An autoclavable μ-milling device has been introduced to contour and drill the lamina to increase its stability (86). Iyer et al. introduced a new technique that augments the canine tooth using a mandibular bone graft to boost the labial side of the lamina and therefore decrease laminar resorption (87). In a separate study, Iyer et al. administered a bone morphogenetic protein to 11 eyes with laminar resorption and yet to undergo additional intervention (88). Bone morphogenetic proteins were administered to inhibit further resorption and promote bone generation. However, three eyes had further resorption after protein administration (88). There is uncertainty around the ability of bisphosphonate drugs such as alendronate to decelerate laminar resorption. Several remedies are available that maintain mucosal health and in addition, smoking cessation can have an increased benefit (85).

To decrease laminar resorption, and simplify the surgical procedure, decrease costs, and avoid oral trauma, skirts made of synthetic materials similar to the osteo-odonto-acrylic lamina have been introduced. Avadhanam et al. incorporated nano-crystalline hydroxyapatite (nHAp) coated poly (lactic-co-glycolic acid) PLGA microspheres with a high strength interpenetrating network (IPN) hydrogel to mimic the odonto-acrylic lamina microenvironment (89). They also added poly(ethylene glycol) diacrylate (PEGDA) polymers and agarose to improve the mechanical strength of the hydrogels. This study suggested the PEGDA-agarose based IPN can be used in the future to replace the OOKP lamina (89).

There is a strong correlation between laminar resorption and endophthalmitis, a condition that is caused by a bacterial or fungal infection of the vitreous and/or aqueous humour (90). A recent study found a 9% incidence rate of endophthalmitis in eyes that had undergone OOKP surgery (91). Falcinelli et al. identified endophthalmitis in 4 out of 181 eyes (2%) following OOKP at a mean 12 years follow-up. Poor pre-operative dental hygiene was reported in these cases (39).

Two common causes of the slowdown in the rate of VA recovery are the presence of air bubbles in the vitreous humour or vitreous haemorrhage. Vitreous haemorrhage was the most common post-operative complication in the systematic review reported by Tan et al., with up to 52% experiencing haemorrhage (80). However, vitreous haemorrhage, and also the problem of choroidal detachment, tend to resolve themselves soon after surgery.

Glaucoma is the main cause of a decrease in VA for those with an OOKP. Tan et al. stated glaucoma rates ranged from 7 to 47% between different studies (80). However, pre-existing glaucoma can be hard to detect pre-operatively (76). Generally, those with glaucoma undergo a trabeculectomy to relieve IOP; however, those with an OOKP will not benefit from this procedure. Kumar et al. found visual field testing and optic disc assessment with optic disc photographs may be used for the monitoring of eyes for glaucoma; but currently, drainage devices are the best method for glaucoma management in those with OOKPs (92, 93). Interestingly, a device called the Ahmed glaucoma drainage device was found to stabilise IOP in three-quarters of OOKP eyes with glaucoma if placed before the mucosal graft (93).

## Soft Keratoprostheses

Soft skirt materials have been adopted in recent years to increase biointegration based on a variety of synthetic polymers with or without biofunctionalisation with macromolecules (Table 3). Several skirt and optic type KPros have been brought to clinical trials over the decades, including the Keraklear (94), the MIRO^®^ Cornea (95), the Legeais BioKpro III (96) and the Korea Seoul-type KPro (99, 100). However, for the purpose of this review, we will focus on the AlphaCor™ keratoprosthesis, the first soft KPro to obtain FDA approval almost 20 years ago, while mentioning the two newest soft synthetic KPros that have begun clinical trials in the last year (CorNeat, and EndoArt).

**Table 3:** Description of commercial Soft-Keratoprostheses with skirts based on soft materials.

**Keratoprosthesis**	**Materials**	**Schematic**	**References**
Keraklear artificial cornea	PMMA + (polyethylene glycol) PEG	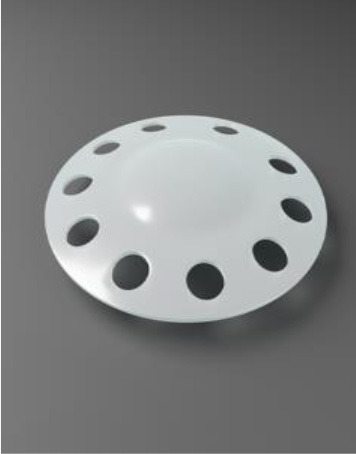	(94)
MIRO^®^ CORNEA UR keratoprosthesis	Hydrophobic acrylic polymer + Genetically engineered fibronectin	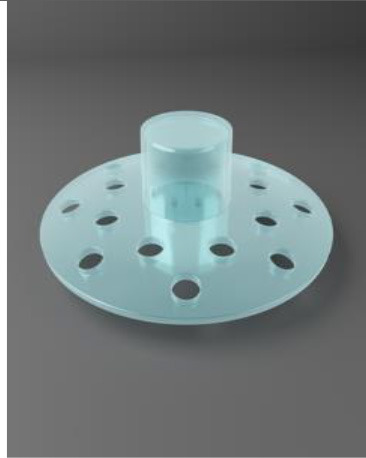	(95)
Legeais BioKpro III	Fluorocarbon poly(tetrafluoroethylene) (PTFE)	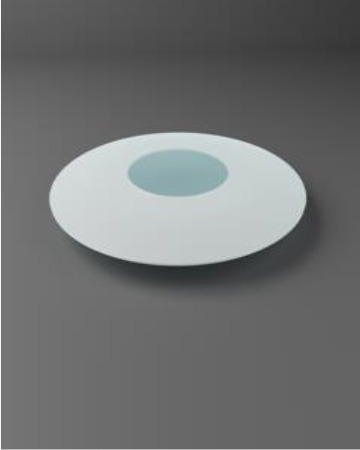	(96)
Alphacor keratoprosthesis	(Polyhydroxyethyl methacrylate) PHEMA	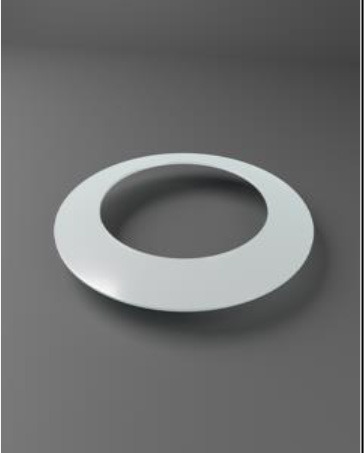	(97, 98)
Korea Seoul-type keratoprosthesis	PMMA + PEG	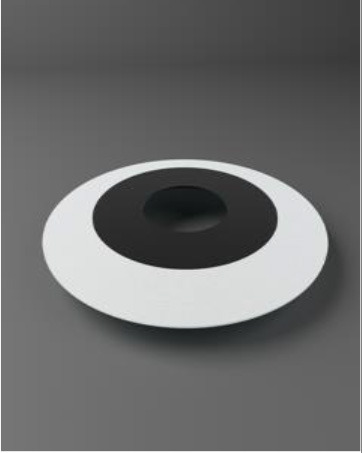	(99, 100)

### AlphaCor™ Keratoprosthesis

**Figure 2:**
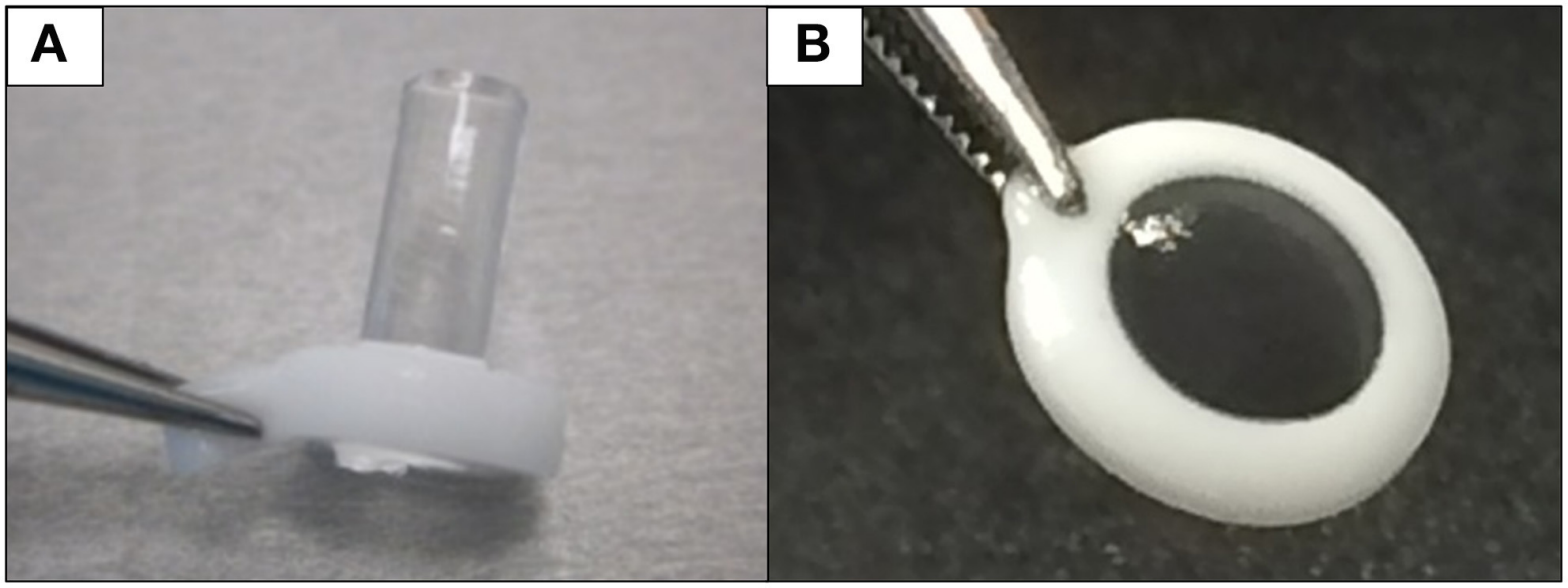
Digital pictures of the AlphaCor™ produced by polymerisation of PHEMA to produce a transparent (optical) and white (skirt) PHEMA before **(A)** and after **(B)** lathing.

Chirila et al. at the Lions Eye Institute and the University of Western Australia in Perth aimed to produce an “ideal” KPro. They used cross-linked poly (2-hydroxyethyl methacrylate) (PHEMA), to form both the optical and skirt components (101). The hydrophilic PHEMA forms a hydrogel by polymerisation. The skirt and optic are chemically identical with the exception that the skirt has higher water content, meaning it has larger pores to allow for biointegration. The optic and skirt are fused by an IPN to prevent leakage or down growth (102, 103). Formerly known as the Chirila KPro, the AlphaCor™ KPro (Figure 2) was approved by the FDA in 2003 (97). In 1998, the original Chirila Type-I KPro was first implanted in three people who had failed keratoplasty and had vascularised and/or scarred corneas. This device required full-thickness removal of the host cornea, and the placement of a conjunctival flap to protect the KPro-corneal interface during post-operative healing; this flap is then removed in a second surgical stage. Unfortunately, two devices were quickly extruded due to retraction of the conjunctival flap. Full-thickness insertion increased the risk of exposing the porous skirt after conjunctival flap failure. In response to these observations, a thinner KPro (the Type II, AlphaCor™ KPro) was developed, allowing for lamellar pocket implantation instead of full-thickness insertion, followed by a subsequent second surgical step several weeks later to trephine the anterior host cornea. A pilot human trial in four patients implanted with the type II KPro observed no post-operative complications, and improved outcomes at seven months follow-up in all individuals (104). This thinner design was subsequently utilised in the larger clinical trials that supported the FDA approval of the AlphaCor™ device (103).

#### Outcomes

Retention rates reported for the AlphaCor™ have been relatively high. In a phase I trial, 93% of the 14 devices were retained for up to 2.5 years (105). Some years thereafter, Hicks et al. undertook a retrospective study of 322 AlphaCor™ KPros and found at 6 months, 1, and 2 years, 92, 80, and 62% of devices were retained, respectively (97). Of 322 AlphaCor™ KPros implanted, 65.8% were *in situ*, 26.7% had undergone a PK, 6.2% had been replaced with a second AlphaCor™, and 1.2% of patients had lost the eye. Stromal melts occurred in 27% of the cases, from which 65% had resulted in device expulsion (97). Similarly, Jiraskova et al. recorded survival rates of 87, 58, and 42% after 1, 2, and 3 years, respectively (98). Conversely, they found stromal melts occurred in 60% of patients and device removal was necessary in more than half of these patients (98). Topical administration of medroxyprogesterone appeared to protect against melts. On the other hand, protection such as bandage contact lenses could have contributed to this decrease in corneal melts (103).

The high water content and therefore large pores of the AlphaCor™ can lead to inadequate suturing performance and overall poor mechanical strength which causes stromal melts and therefore extrusion. A T-style KPro based on a PHEMA hydrogel was introduced by Xiang et al. to address this problem. They found that adding hyaluronic acid and cationised gelatin to the skirt promoted cell adhesion and bound the device and native tissue firmly (106). They also added poly(ethylene glycol) (PEG) to the bottom of the optical column and this caused resistance to RPM formation by decreasing cellular attachment and proliferation (106).

Often patients will have pre-existing conditions such as macular disease or glaucomatous cupping which will limit VA improvement. This was the case for many patients in the study carried out by Hicks et al. The average VA was 20/200 and the lowest was light perception. Surprisingly, one patient did achieve a VA of 20/20 (97). Jiraskova et al. found BCVAs ranging from hand movements to 20/25 (98). Although there are promising visual acuity results, regain of sight was often impeded by the occurrence of deposits on the optic and surface spoliation of the device. Hicks et al. found that 11% of all patients implanted with an AlphaCor™ had intraoptic calcium or pigment deposition, four cases having white deposition and the other four brown (107). The white deposits had been associated with topical steroid and beta-blocker administration and the brown deposits were correlated with cigarette smoking and topical administration of the beta-blocker levobunolol (107). Interestingly, one study excluded stage 2 of the surgical process, in which corneal tissue is removed from the anterior flap, to find if this could decrease the rate of stromal melts, deposits, and aqueous leakage. All six patients had no stromal melting, infection, aqueous leakage, or extrusion (108).

The AlphaCor™ was developed to address the problems observed in older generations of KPros; namely glaucoma, endophthalmitis, RPM formation, and extrusion. The AlphaCor™ is associated with reduced complications, but corneal stromal melts and optic deposition have been a major setback. Current efforts to improve the clinical outcome include enhancing the stiffness of the skirt material to allow for better suturing of the device into the host eye. Furthermore, the incorporation of gelatin to improve cell attachment and proliferation are also under consideration to enhance device skirt biointegration. In addition, efforts are being made to improve the optics of the AlphaCor™, such as the addition of a UV philtre co-monomer to avoid any UV-associated damage to the retina, as well as the use of an anti-calcification comonomer to reduce the risk of optic depositions that impair visual improvements. It is hoped that these modifications will target the majority of complications previously identified with AlphaCor™, and that future patients will benefit from these improved clinical outcomes.

### Synthetic Cornea Alternatives

An interesting alternative to the AlphaCor™ is the CorNeat KPro, a completely synthetic, sterile cornea made using inert materials. Whereas, the AlphaCor™ attempts to somewhat biointegrate with native tissue (stroma), the tissue itself is avascular and is slow to heal. The CorNeat KPro takes advantage of the highly vascularised, fibroblast rich, regenerative environment of the conjunctiva and biointegrates with the tissue.

In contrast to the lengthy MOOKP surgical technique, CorNeat implantation requires just 45 min of surgery using a surgical kit with a marker and snapper. The PMMA lens is designed to effortlessly snap on into a trephined cornea. If successful, the device should withstand IOP and uphold the eye's integrity. The degradable skirt is implanted subconjunctivally.

The CorNeat KPro is indicated for those who have had keratoplasty failure or an indication that would result in keratoplasty failure (109). The CorNeat has just entered its first-in-human clinical trial in Israel as of January 2021 (ClinicalTrials.gov Identifier: NCT04485858). Several other clinical trials are planned and have a predicted release date of 2023. Moreover, a synthetic endothelial layer has been produced by an Israeli company, EyeYon Medical, known as EndoArt (110). It is a polymer film that acts as a barrier, preventing excess fluid from entering the cornea from the anterior chamber, thereby avoiding corneal oedema and vision loss. The EndoArt is implanted by a minimally-invasive procedure and can reduce pre-existing edoema, as evident in pre-clinical studies and in an early clinical trial (ClinicalTrials.gov Identifier: NCT03069521).

## Future Trends for Corneal Implants

In contrast to KPros, a growing area of research and development relates to corneal substitutes aimed at reducing reliance on human donor tissue, in particular for the low-risk cases comprising the majority of corneal transplantations performed worldwide. Various biomaterials have been employed to form full- or partial-thickness corneal substitutes to replicate the structure and function of the cornea. Both natural and synthetic polymers have been used as scaffolds and substitutes for corneal stroma (111). Natural polymers have the advantage of biocompatibility, but synthetic polymers allow for manipulation of chemical and mechanical properties to meet individual needs (112).

Biopolymers of extracellular matrix (ECM) components are being investigated to mimic the corneal microenvironment. In theory, ECM components should be ideal for promoting and supporting regeneration as it is the ECM that supports the growth and embryonic development of an organ. The ideal biomaterial should be biocompatible, transparent, strong (to allow suturing and IOP), non-immunogenic, refractive, permeable to nutrients and oxygen, and resistant to neo-angiogenesis (113).

### Collagen and Derivatives

The corneal stroma, which makes up the bulk of the cornea, consists mainly of collagen. Collagen type-I is abundant in several areas of the body, and it is commercially available (112). In the cornea, collagen type-I, III and V form a complex lattice-like structure that provides considerable strength, but this is difficult to replicate in a laboratory setting using purified collagen from different species and tissues. Several treatments have been applied to collagen hydrogels to increase their tensile strength (113). Collagen hydrogels have been plastically compressed to increase density (114), cross-linked chemically (glutaraldehyde, genipin), physically (UV or dehydrothermal treatment) or enzymatically (transglutaminase) (112), and added to other materials capable of forming an IPN or double network (115).

One promising solution for patients with a high-risk of graft failure is a bioengineered corneal implant made from recombinant human collagen type III (RHCIII). In a phase I clinical study, Fagerholm et al. prepared a biosynthetic cornea composed of type III recombinand human collagen crosslinked with the non-toxic zero-length crosslinkers 1-ethyl-3-(3-dimethylamino-propyl)-carbodiimide hydrochloride and N-hydroxysuccinimide (EDC-NHS). It was found that the biomimetic cornea had good biointegration, regenerated the corneal epithelium, partially replaced the corneal stroma and facilitated nerve regeneration, that restored the corneal reflex better than corneal allografts in low-risk patients (116). A 4-year follow-up showed all 10 implants maintained their transparency and no tissue rejection was reported (117). However, these RHCIII implants were only suitable for low-risk patients as they led to neovascularisation in rabbit models with severe pathology (118).

To identify whether the risk of implant-related neovascularisation in high-risk patients could be reduced, modified RHCIII implants were developed to include the synthetic phospholipid methacryloyloxyethyl phosphorylcholine (MPC). These RHCIII-MPC implants had previously been shown to prevent vascularisation in a high-risk alkali burn corneal injury model (118). This device was implanted into three patients with ulceration, decreased corneal integrity, near blindness and associated pain and discomfort (119). Although the implants improved vision in only two of the three patients, in all three cases, the implants remained free of neovascularisation at 1-year follow-up. Functional restoration of corneal integrity was observed, with stable regeneration of both the corneal epithelium and nerves, providing all three patients relief from pain and discomfort (119).

In 2018, Islam et al. grafted cell-free corneal implants consisting of recombinant human collagen and MPC by anterior lamellar keratoplasty (120). The patients were unilaterally blind and at high-risk of graft failure. Three out of six patients gained significant improvement in vision and the corneal stability of the remaining patients was sufficient to allow surgery to improve vision. Grafting outcomes in mini-pig corneas were superior to those in human subjects, indicating that animal models are only predictive for patients with non-severely pathological corneas (120). Another method to combat neovascularisation is to integrate a sustained release nanosystem of bevacizumab (an anti-VEGF drug) into the cell-free biosynthetic scaffolds (121), while ulceration and a neurotrophic deficit could be addressed by sustained release of nerve growth factor, demonstrated recently in a collagen-based scaffold releasing the drug in a controlled manner during a 60-day period (122).

Limbal epithelial stem cells (LESCs) have been successfully cultivated on recombinant human collagen type-I (RHCI) hydrogels (123). LESCs at the junction of the sclera and cornea are responsible for the regeneration of corneal epithelial cells and also prevent invasion by conjunctival epithelial cells (124). Severe limbal stem cell deficiency requires keratolimbal and limbal stem cell allografts but these have poor survival rates and usually require immunosuppression post-surgery (125, 126). One study found that the LESC cultivated hydrogels were biocompatible, had promising optical characteristics, comparative microbial resistance and successful composite graft generation (123). Additionally, human corneal stromal-derived mesenchymal stromal cells (MSCs) have been shown to culture successfully on a porcine collagen-based hydrogel scaffolds (127).

In 2020, McTiernan et al. introduced the LiQD cornea. The LiQD cornea is made up of short collagen-like peptides conjugated with PEG which are functionally similar to RHCIII implants (128). Fibrinogen was also added to act as a natural adhesive. The LiQD Cornea is liquid at temperatures above 37°C and solidifies to a gel at lower temperatures. It therefore can be used as either a sealant or an alternative to corneal transplants. A 12-month study carried out on pigs found the cornea capable of regeneration and a reduced risk of allergy or immune reaction was observed in traditional corneal transplants or xenogeneic materials, however, all implanted pigs had corneal haze and neovascularisation post-operatively (128).

Alternatively, gelatin, a denatured form of collagen, can be used to construct membranes for corneal cells. It is more pre-disposed to biodegradation and absorption than collagen itself. Gelatin can be cross-linked dehydrothermally or chemically using EDC or glutaraldehyde (GA). Mimura et al. cross-linked a gelatin hydrogel with GA and found the hydrogel was capable of supporting the growth and maintenance of cultured rabbit fibroblasts for 4 weeks (129).

Several other materials beyond collagen or gelatin, such as silk and chitosan are now being investigated to form corneal substitutes with some success (130). This ever-growing area of research has the potential of forming full-thickness corneal biomimetic substitutes in the future.

### Decellularised Corneas

Decellularised corneas are one of the most promising forms of replicating the complex structure and function of actual corneas (111, 131). Decellularisation is a process by which cells from mammalian organs or tissues are removed to form a cell-free scaffold with intact ECM integrity. Although hydrogels derived from ECM components such as collagen mimic the cornea's ECM, they may lack its fibril organisation (127) and thereby the tensile strength that the lamellar collagen structure imparts to the stroma.

Decellularised corneas mimic both the fibril architecture and corneal composition and therefore, are a very attractive option. It is the organised and complex architectural structure of the stromal collagen fibrils in the cornea that allow for the appropriate biomechanical properties of the cornea. Collagen fibrils in the anterior part of the cornea are more isotropic and thus allow the IOP to be better withstood and to sustain corneal curvature. Here, spring-like structures extend into deeper fibrils (132). Peripherally the fibrils are circumferentially orientated, more compact and the fibril diameter increases with the merging sclera collagen to reinforce the limbus stabilising the corneal curvature and sustaining its refractive properties (133). The larger and wider fibrils of the posterior cornea and their orthogonal arrangement, as well as the ones of the central cornea, strengthens against strain from extraocular muscles. Narrower bundles in the posterior stroma are directed to the four major rectus muscles. This complex collagen structure is maintained by proteoglycans and glycosaminoglycans. Decellularised corneas are a promising source for engineering corneal tissue as they retain this complex structure of corneal collagen (132).

The process of decellularisation starts with the isolation of the donor tissue followed by the removal of the cells. New healthy cells can then be added to increase biointegration and finally, the cornea is implanted into the patient (131). Decellularisation can be achieved using physical (freeze-thaw cycle, high hydrostatic pressure, electrophoresis, supercritical CO_2_), chemical (Triton X-100, sodium dodecyl sulphate, formic acid, ethanol) and/or biological agents (trypsin, phospholipase A2, Dispase^®^ II). Decellularisation aims to eliminate from the cornea all major histocompatibility complexes to prevent an immune response and therefore rejection once transplanted into the recipient (131). It has been shown that ineffective decellularisation causes macrophages to change into their pro-inflammatory M1 phenotype *in vivo* and *in vitro* (134). Moreover, decellularisation may expose new antigenic sites due to the deformation of the collagen fibrils, which may lead to graft rejection (135). In addition, the process of decellularisation often significantly reduces proteoglycan content. This reduction in proteoglycan content reduces the water holding capacity of these constructs and compromises bioactivity.

Porcine corneas are commonly used for decellularisation studies as they are easily procured and have anatomical similarities with the human cornea. In the case of porcine cells, decellularisation is necessary to eliminate the epitopes Galactose-alpha-1,3-galactose (α-Gal) and N-glycolylneuraminic acid (Neu5Gc) which are extremely immunogenic to human hosts (136). Suboptimal decellularisation procedures leading to immunogenic reactions are likely the source of inflammation, neovascularization and rejection observed in the first clinical reports of acellular porcine corneas implantation (137).

To address potential issues of xenogeneic transplantation, a potential alternative is to first generate “humanised” pigs. To develop “humanised pigs” one must remove multiple xenoreactive cell surface molecules and porcine endogenous retroviruses (PERV). The revolutionary CRISPR-Cas9 gene-editing technique has been introduced to obtain pigs with GGTA1, CMAH and β4GalNT2 gene knockouts involved in immunogenic surface glycans (138). PERVS were also inactivated using the same technique (139), possibly making the corneas of transgenic pigs a non-immunogenic alternative. However, the process is very costly compared to using decellularised corneas from “normal” pigs.

Following decellularisation, these matrices may be populated with human cells to generate a viable corneal transplant. There are three parameters used to establish that decellularisation has taken place: staining to verify the absence of intact cell nuclei, quantification of double-stranded DNA (dsDNA), and determination of the maximum length of DNA remnants using agarose gel electrophoresis. The difficulty in choosing the optimal decellularisation technique lies in the fact that researchers have obtained different results using similar techniques. Also increasing decellularisation efficiency is associated with increased damage to the ECM (131).

Recellularisation of the cornea can be achieved using cells from many different origins, all of which are associated with certain advantages and disadvantages. As the cornea is avascular, allogenic cells can be used with a decreased risk of rejection, provided the implanted tissue remains sequestered from the host immune system. Recellularisation of the three different cell types- epithelium, stroma and endothelium- has been carried out using different approaches.

Recellularisation of the stroma is possible using autologous stromal cells by obtaining a biopsy from the uncompromised eye. If both eyes are compromised, adipose-derived MSCs can be activated to produce keratocytes (140). Induced pluripotent stem cells (iPSCs) cultured on cadaveric human corneas have produced cells with a similar phenotype to keratocytes (140).

Different means of achieving cell penetration into the thick densely packed fibril structure of the cornea have been investigated. Human keratocytes seeded directly onto the surface of the scaffold have resulted in distributions resembling human counterparts. In a phase I clinical trial, Ali del Barrio et al. successfully recellularised 120 μm thick laminas from donor corneas by seeding autologous adipose-derived MSCs which were implanted in four patients (141). Each patient had an improvement in VA and CDVA. However, there was no significant difference between the recellularised and non-recellularised groups, questioning the need to add adipose-derived MSCs, which are obtained from an extra liposuction surgery (141).

Injections of cells into the stroma can damage the stromal fibril structure (131). Freeze-drying creates pores which allow for greater cell penetration (142). Bioreactors have been used where the construct is kept in suspension using a magnetic stirrer and cells, prevented from attaching to other surfaces and promoting the colonisation of the structure. Ma et al. seeded thin sheets of decellularised porcine cornea with keratocytes during transplantation. Cells were added to each sheet, creating a 5-layer recellularised cornea which was then transplanted into rabbits by lamellar keratoplasty (143). Surgery using these recellularised sheets was more successful and had greater transparency than surgeries involving acellular tissue in the model (143).

Epithelial recellularisation was carried out using limbal stem cells isolated from a biopsy of the unaffected eye (144). When both eyes are compromised, oral mucosal allogeneic cells can be used (145). iPSCs could also be used as a non-autogenic cell source due to their ability to differentiate into limbal epithelial stem cell-like cells (146). Xu et al. reported the production of an anterior hemi-cornea using acellular porcine corneal stromata injected with human corneal stromal and epithelial cells (147). These constructs were transfected into dog eyes by lamellar keratoplasty and found to maintain corneal transparency, thickness, and composition (147).

Native corneal endothelial cells are arrested in the G_1_ phase, and therefore will not proliferate. However, *in vitro* endothelial cells can proliferate but procedures must be established to restrict the cells from transitioning into MSCs (131). The use of these cells relies on donor corneas. iPSCs can form human corneal endothelial-like cells which can potentially be used for implantation (148). Choi et al. reported the dissection and sectioning of donor corneal stroma to 120–200 mm thick (149). Following decellularisation, these stromal sections were seeded with human donor-derived corneal endothelial cells which resulted in a neo-cornea with biomechanical properties comparable to a normal cornea after 14 days in culture (149).

Some reports have questioned the merits of recellularisation as no significant difference had been observed between the acellular and recellularised corneas (141). However, this is a developing field that requires more *in vivo* studies and clinical trials to assess the possible advantages of recellularisation. Nevertheless, decellularised corneas could provide a potential cornea alternative that mimics both its composition as well as its fibril architecture.

### 3D Bioprinting

3D printing has become an attractive method to manufacture a corneal equivalent. With the emergence of various biomaterials in corneal bioengineering, bio-inks and inks can be made to mimic the corneal microenvironment. Currently, much of the emphasis is on rebuilding a stromal equivalent using several methods which include inkjet printing, extrusion printing, and Laser-assisted printing (8). Duarte Campos et al. (150) bioprinted corneal stromal keratocytes (CSK) in collagen-based bioinks to form stromal equivalents. Theoretically, 3D bioprinting could produce a multi-layered cornea embedded with epithelial cells, keratocytes and endothelial cells.

Isaacson et al. (151) demonstrated the feasibility of engineering an artificial corneal structure using 3D bioprinting. Using an existing 3D digital human corneal model and a composite bio-ink comprising of collagen and alginate, which contained encapsulated corneal keratocytes, 3D constructs anatomically analogous to a human model were produced (151). Keratocytes remained viable for 7 days post-printing. However, the metabolic activity and the protein expression of the keratocyte cells was low which might be linked to the high crosslinking density of the 3D bioprinted scaffold and the lack of a curved geometry (151).

Ulag et al. have 3D printed a cornea suitable for transplantation using an aluminium mould, necessary to achieve the correct shape and a PVA-chitosan construct (152). Scanning electron microscopy and UV spectrometry showed favourable optical properties. Tensile strength could support fluctuations in IOP and the structure remained biocompatible with stem cells after 30 days of degradation (152).

Moreover, decellularised corneal ECM-based bio-inks can be used to mimic the corneal stroma structure. Kim et al. investigated the effects of changing the nozzle diameter and hence the shear stress when extrusion bioprinting was used to bio-print human keratocytes into a bio-ink made from decellularised corneal ECM (153). Widening the nozzle to lower shear stress resulted in non-aligned collagen fibrils. While giving highly structured fibrils, the narrower nozzle and higher shear stress damaged the keratocytes, thereby activating fibroblasts. Finally, the optimal nozzle diameter produced a structure similar to the native human corneal stroma with viable keratocytes (153).

Sorkio et al. produced a scaffold containing a stromal layer and an epithelial layer using laser-assisted bioprinting (154). The epithelial layer was created using a bio-ink containing human recombinant laminin, hyaluronic acid, and human embryonic stem cells-derived LESCs. The stromal layer was printed with a bio-ink comprised of collagen type 1, blood plasma, thrombin and human adipose tissue-derived stem cells. The structure mimicked the human corneal stroma and supported high cellular viability, but the scaffold lost its shape after a few days. In addition, the supporting membrane added to support the stromal layer led to opacity, rendering the structure non-functional (154).

Finally, Kim et al. bioprinted a scaffold using a gelatin ink in which human corneal endothelial cells were embedded. These cells had been genetically modified to express ribonuclease 5 (R5) which increases endothelial cell proliferation (155). The scaffolds showed transparency and cell viability, and 4 weeks after transplantation of the 3D structures to rabbit corneas, this group showed better transparency than the non-printed group (155). Even though the majority of research focuses on manufacturing a stromal equivalent, 3D bioprinting does have to potential to form a full-thickness, multi-layered cornea model in the future.

## Conclusions

Artificial corneas range from KPros with biological interfaces for treating intractable cases where donor corneas fail, to cell-free medical devices intended to be a primary replacement for donor corneas. The focus of this review was the evolution of KPros and recent developments in corneal substitutes. The Boston KPro and OOKP have stood the test of time by adapting to arising complications. In terms of the Boston KPro, many changes in its design have been implemented to address certain problems: (i) holes were added to the backplate for nutritional support which significantly reduced keratolysis, (ii) titanium sputtering has been introduced to increase PMMA and corneal tissue adhesion, (iii) pressure sensors were investigated to prevent *de-novo* glaucoma, (iv) titanium backplates and a threadless design have shown potential in decreasing RPM formation, and (v) electrochemical anodisation can colour the titanium backplates blue or brown to increase its aesthetic appeal.

In comparison to the Boston KPro, the OOKP has had limited modifications since its creation in 1963, but there have still been several advancements in the surgical procedure involved. Since alterations to the surgical procedure, introduced by Falcinelli, the OOKP has provided the best visual outcomes of any KPro. Some studies have attempted to address the frequent laminar resorption observed with the OOKP, by using an autoclavable μ-milling device, bone morphogenetic proteins, bisphosphonate drugs and/or remedies to maintain mucosal health. The OOKP serves patients with different indications to the Boston KPro. In general, the Boston KPro is for patients with wet, blinking eyes while the OOKP is for patients with dry, non-blinking eyes.

To address the problems of the previous generations of KPros (namely *de-novo* glaucoma, endophthalmitis, RPM formation and extrusion), the AlphaCor™ was developed. This PHEMA-hydrogel-based KPro significantly reduced these complications; however, other complications arose, such as the occurrence of corneal stromal melts and optic deposits, which have greatly curtailed its use. Some hope comes in the form of synthetic corneas such as the CorNeat which have the potential to completely integrate into the native tissue by joining the conjunctiva, improving both the aesthetic appeal and incidence rate of complications associated with artificial corneas. Nevertheless, the CorNeat has only begun clinical trials.

Although these approaches have focused mainly on artificial corneas made of synthetic materials, much of the interest now lies in using naturally occurring matrix macromolecules such as collagen to form scaffolds for tissue reconstruction and/or delivery of cell-based therapies. These technologies have the advantage of potentially addressing the much larger group of low- to medium-risk indications for corneal transplantation, in contrast to KPros. Decellularised corneas have a potential although a multi-layered corneal alternative and recellularisation using the three corneal cell types has yet to be accomplished. In contrast to biomaterials-based scaffolds, decellularised corneas mimic the complex corneal fibril architecture. However, the immune response to these decellularised constructs is not yet fully understood and initial clinical outcomes have been suboptimal.

First conceived in 1789, artificial corneas have come a long way- from a rudimentary quartz crystal implanted in rabbit eyes to a fully functional, full-thickness KPro implanted in thousands of eyes. Albeit only specified for those who have, or will fail, corneal transplantation, artificial corneas have restored sight to many blind patients. Furthermore, constant improvements in the design have greatly impacted the rate of complications such as RPM formation, glaucoma and endophthalmitis. Soft KPros have demonstrated enormous clinical potential; however, the use of certain biomaterials as components in polymer-based synthetic corneas or 3D printed structures, and the development of decellularised corneas have still presented with serious complications. Concentrated efforts towards improving the biointegration and reducing complications of biofunctionalised soft Kpros may hopefully lead to a successful artificial cornea in the near future.

## Author's Note

KPros in general, and particularly the Boston KPro, have shown very good results for vision rehabilitation in eyes where penetrating keratoplasties have failed and where the eye is not severely inflamed. If high levels of inflammation such as in immune disorders (like MMP or SJS) or severe chemical burns are present, however, the KPros are likely to extrude. Drawbacks also include complications such as RPM formation and glaucoma, and KPros often require multiple surgical interventions. A human corneal button is also needed for the implantation, so the KPro does not address the donor cornea shortage, while the expense of the KPro and associated procedures render it unaffordable in many countries. Nonetheless, it should be kept in mind that despite numerous difficulties and limitations, KPros have restored sight and quality of life to thousands of patients worldwide and continue to do so today.

As KPro outcomes are typically poorer in in inflamed eyes, there is a clear need to develop devices and/or protocols to better control the inflammation (e.g., biologics), to prevent extrusion, and improve the retention rate. Here, technologies for sustained release of drugs, either integrated within the KPro itself or implanted within the eye at the time of surgery, could improve outcomes and reduce post-operative complications. Likewise, wireless, or remote monitoring of IOP could aid in the post-operative management of glaucoma.

Although the “classic” KPros will still play an important role in the immediate future for the treatment of serious ocular disorders in high-risk eyes, less technically challenging KPros such as the AlphaCor™ KPro might have an advantage if biointegration can be improved and extrusion can be prevented. We feel, however, that advances in materials, coatings, drug delivery, and 3D (bio)printing could enable a newer generation of KPros to be developed which overcome current limitations. Regarding lower risk eyes where donor corneas could be used if available, several promising approaches exist, although these are still in the development phase. Decellularisation and recellularisation of corneal tissue, from human or non-human sources must still overcome the potential for immunogenicity, and here immunomodulatory cells such as MSCs could play a role. Nonetheless, using intact corneal tissue does not allow for complete control over corneal properties. For this, technologies such as bioprinting and other forms of laboratory-made corneas offer the ability to design a cornea from the “ground up” by choosing the ECM, cell types, and other factors as well as maintaining control over their spatial organisation. This flexibility may prove advantageous, particularly for niche indications. This field, however, is still nascent and very much in an exploratory research phase.

In the more distant future, induced pluripotent stem cell (iPSC) technology might it make possible to generate human eye organoids *in vitro*, for subsequent transplantation into diseased eyes. However, it remains to be seen how these *in vitro* generated corneal transplants will fare in very diseased human eyes, although, where feasible, an autologous source for iPSCs would render the organoids perfectly immune-compatible. In cases where genetic deficiencies exist, allogeneic iPSC-derived tissues could be tolerated by applying Crispr/Cas gene technology to yield MHC-deficient corneal organoids as a universal source for low-risk corneal transplants.

## Author Contributions

GH, EM, and TR wrote the manuscript. AP, LS-A, AH, IL, DD, MG, EL, AB, and NL contributed to specific parts of the review and revised and approved the final version of the manuscript. All authors contributed to the article and approved the submitted version.

## Funding

This project has received funding from the European Union's Horizon 2020 research and innovation programme under Grant Agreement No. 814439. The authors also wish to acknowledge the support of the European Union COST Programme under COST Action CA-18116, ANIRIDIA-NET. In addition, the authors acknowledge the support of Grant Number 13/RC/2073_P2, a research grant from Science Foundation Ireland (SFI) cofunded under the European Regional Development Fund.

## Conflict of Interest

The authors declare that the research was conducted in the absence of any commercial or financial relationships that could be construed as a potential conflict of interest.

## Publisher's Note

All claims expressed in this article are solely those of the authors and do not necessarily represent those of their affiliated organizations, or those of the publisher, the editors and the reviewers. Any product that may be evaluated in this article, or claim that may be made by its manufacturer, is not guaranteed or endorsed by the publisher.

## Abbreviations

3D: Three-dimensional
ACGR: Australian corneal graft registry
ALK: Anterior lamellar keratoplasty
BCVA: Best-corrected visual acuity
BM: Buccal mucosa
CaP: Calcium phosphate
CDVA: Corrected distance visual acuity
CSK: Corneal stromal keratocytes
DALK: Deep anterior lamellar keratoplasty
DM: Descemet membrane
DMEK: Descemet's membrane endothelial keratoplasty
DSAEK: Descemet's stripping automated endothelial keratoplasty
dsDNA: Double-stranded DNA
ECM: Extracellular matrix
EDC: 1-Ethyl-3-(3-dimethylamino-propyl)-carbodiimide hydrochloride
EK: Endothelial keratoplasty
GA: Glutaraldehyde
HAp: Hydroxyapatite
IOP: Intraocular pressure
IPN: Interpenetrating network
iPSC: Induced pluripotent stem cells
KPro: Keratoprosthesis
L-DOPA, L-3: 4-Dihydroxyphenylalanine
LESC: Limbal epithelial stem cell
LK: Lamellar keratoplasty
MDCT/CT: Multi-detector computerised tomography
MMP: Mucous membrane pemphigoid
MOOKP: Modified osteo-odonto-keratoprosthesis
MPC: 2-methacryloyloxyethyl phosphorylcholine
MSC: Mesenchymal stromal cell
Neu5Gc: N-Glycolylneuraminic acid
nHAp: Nano-crystalline hydroxyapatite
OCT: Optical coherence tomography
OOKP: Osteo-odonto-keratoprosthesis
PEG: Polyethylene glycol
PEGDA: Poly(ethylene glycol) diacrylate
PERV: Porcine endogenous retroviruses
PHEMA: Polyhydroxyethyl methacrylate
PK/PKP: Penetrating keratoplasty
PLGA: Poly (lactic-co-glycolic acid)
PMMA: Poly (methyl methacrylate)
PTFE: Poly(tetrafluoroethylene)
RPM: Retroprosthetic membrane
SALK: Superficial anterior lamellar keratoplasty
SJS: Stevens-Johnson syndrome
VA: Visual acuity
α-Gal, Galactose-alpha-1: 3-galactose.
